# East Suffolk and Ipswich Hospital

**Published:** 1914-08-01

**Authors:** 


### East Suffolk and Ipswich Hospital.

The seventy-eighth Court of Governors of this hospital was held on July 24, Mr. H. W. Mason in the chair. The Court was largely attended, and many workpeople's representatives were present.

Sir Henry Burdett's Report on this institution (see The Hospital, December 6, 1913) contained many recommendations which have been taken up by the board, who have entered upon a progressive policy which has led already to an improvement in the financial position of the hospital. The board recognised the force of Sir Henry Burdett's recommendation that the income should be increased by £1,500 to £2,000 a year, and has set to work with a will to secure this additional revenue. At the request of the board, the secretary brought up a report which contained numerous suggestions for augmenting the revenue, of which seventeen were adopted. Various faults in the administration which Sir Henry Burdett pointed out have been under the consideration of the board; and some of them, including the appointment of two anæsthetists, the provision of an honorary assistant bacteriologist, the opening of a new department for vaccine-therapy, and increased salaries and holidays under better conditions, with a widened scope of training for nurses, have already been agreed to. The board has yet to appoint a special committee—a step urgently called for—to examine into and report upon the existing regulations and medical arrangements of the hospital. The pressing needs of (*a*) a semi-convalescent home under the management of this hospital, and (*b*) of balconies to the main wards were emphasised in the report. This report contains gratifying evidence of the progressive spirit now present, and indicates that every community throughout England at the present time can be influenced by this spirit when exhibited by the hospital board. Such a spirit will quicken the public everywhere to give all the funds required by each voluntary hospital, for the people will then recognise that they must rely upon its help in the day of illness or accident. We congratulate the Ipswich Board on being one of the first to present a budget to the governors each year containing an estimate of the expenditure for the forthcoming year. Mr. Mason and Mr. F. Messent, the chairman of the board, both expressed their appreciation of the zeal and energy of their secretary, Mr. Arthur Griffiths, who devotes his whole time and energy to the interests of the hospital.

